# Impact of Reduced Dietary Crude Protein and Propionic Acid Preservation on Intestinal Health and Growth Performance in Post-Weaned Pigs

**DOI:** 10.3390/ani15050702

**Published:** 2025-02-27

**Authors:** Kathryn Ruth Connolly, Torres Sweeney, Marion T. Ryan, Stafford Vigors, John V. O’Doherty

**Affiliations:** 1 School of Agriculture and Food Science, University College Dublin, D04 W6F6 Dublin, Ireland; ruth.connolly@ucdconnect.ie (K.R.C.); staffordvigors1@ucd.ie (S.V.); 2 School of Veterinary Medicine, University College Dublin, D04 W6F6 Dublin, Ireland; torres.sweeney@ucd.ie (T.S.); marion.ryan@ucd.ie (M.T.R.)

**Keywords:** weaning, protein, microbiota, inflammation, organic acid, grain, sustainability, antimicrobials, swine

## Abstract

This study investigated whether organic acid (OA)-preserved grain could mitigate the negative effects of low crude protein (CP) diets on growth performance, intestinal health, and the coefficient of total tract digestibility (CATTD) of nutrients in weaned piglets. The grain was either conventionally dried or preserved post-harvest with 4 kg of OA per tonne. Ninety-six piglets (28 days old) were assigned to one of four diets in a 2 × 2 factorial design: (1) dried standard CP diet, (2) OA-preserved standard CP diet, (3) dried low CP diet, and (4) OA-preserved low CP diet. Standard and low CP diets contained 20% and 19% CP during the first 15 days, reduced to 19% and 17% CP from days 15–35 post-weaning. Faecal scores (FS) were assessed twice a day while microbial composition, inflammatory markers, colonic volatile fatty acid concentrations, and intestinal morphology were measured on the 8th day post-weaning. Performance metrics were measured over the 35-day experimental period. Low CP diets consistently reduced FS (*p* < 0.05) and increased colonic molar butyrate proportions (*p* < 0.01) but increased duodenal *IL1B* expression compared to standard CP diets (*p* < 0.05). The OA-preserved grain enhanced beneficial microbial populations (*Lactobacillus*, *Roseburia*) while lowering pro-inflammatory cytokines (*IL1A*, *IL17*) (*p* < 0.05). While dried grain with low CP diets reduced average daily gain (ADG), colonic short-chain fatty acids (SCFA) concentrations, and nitrogen digestibility, OA-preserved grain with low CP maintained these parameters and improved final body weight (*p* < 0.05). Overall, OA-preserved grain mitigated the performance decline associated with low CP diets by enhancing gut health and nutrient digestibility and reducing inflammation, thus presenting a promising alternative nutritional strategy for post-weaned piglets.

## Simple Summary

This study examined whether grain preserved with organic acids (OAs) could help counteract the negative effects of low crude protein (CP) diets on the growth performance, gut health, and nutrient digestibility of weaned piglets. The grain was either dried using conventional methods or preserved with 4 kg of OA per tonne. Ninety-six piglets, 28 days old, were assigned to one of four diets: (1) dried standard CP, (2) OA-preserved standard CP, (3) dried low CP, and (4) OA-preserved low CP. Standard CP diets contained 20% CP for the first 15 days, reduced to 19% afterwards, while low CP diets contained 19% and 17%, respectively. The study found that the low CP diets improved gut health by reducing faecal scores and increasing beneficial gut bacteria but also increased the duodenal expression of a pro-inflammatory marker. Importantly, piglets fed the OA-preserved grain reduced the expression of intestinal inflammatory markers and had higher levels of beneficial gut bacteria. While low CP diets with dried grain led to poorer growth and nitrogen digestion, piglets fed the OA-preserved grain maintained similar growth and nitrogen digestion as those on standard CP diets. This resulted in heavier body weights and better overall health. In conclusion, OA-preserved grain helped piglets maintain growth performance, improve gut health, and better nutrient digestibility when fed a low CP diet.

## 1. Introduction

Commercial weaning practices precede the complete maturation of the pig digestive tract and associated immune responses [1]. Weaning reduces feed intake [2], impairs intestinal barrier function, increases inflammation [3], and negatively alters the gut microbiome [4]. These stressors combined with the piglet’s immature systems increase pathogen proliferation, reduce growth, and promote post-weaning diarrhoea (PWD) [5]. Zinc oxide (ZnO) and in-feed antimicrobials have successfully reduced PWD in pigs [6]. However, concerns over antibiotic resistance and environmental impact [7] led the EU to ban these in 2022; (Commission Implementing Decision of June 2017, C (2017) 4529 Final; Regulation (EU) June 2019). As a result, new dietary strategies to support gastrointestinal health in piglets are needed.

Weaned pig diets typically require a crude protein (CP) content of 20–23% for growth and development [8]. However, piglets struggle to digest protein due to its high acid-binding capacity and their limited secretion of gastric hydrochloric acid (HCl) and digestive enzymes [9]. Reduced HCl increases pathogen survival [10], while undigested protein ferments in the colon, producing harmful metabolites such as amines and ammonia, contributing to PWD [11]. Piglets are especially vulnerable to PWD during the initial 14 days post-weaning [12]. Although lowering dietary CP reduces diarrhoea [13,14], levels below 20% can impair piglet growth and intestinal health [15,16].

Organic acids (OAs), specifically propionic acid, are highly effective feed preservatives, inhibiting microbial growth while also preserving grain quality [17,18]. In temperate climates, grain moisture can reach 20–25% at harvest [19], thereby optimising conditions for mycotoxin production [20]. To prevent this, moisture levels must be quickly reduced to 12–14% [21], typically through mechanical drying, an energy-intensive process that produces significant emissions [22,23,24]. Organic acid supplementation may improve protein digestion by lowering the gastric pH, stimulating gastric proteases, and reducing the buffering capacity of feed [25]. This may also reduce the amount of undigested protein reaching the colon by increasing protein digestion and increasing nutrient availability for the pig. Additionally, the lower gastric pH associated with OA supplementation reduces the survival of ingested pathogens [9]. However, there is a paucity of research examining the effects of OA-preserved grain on protein utilisation in post-weaned piglets. The OA-based grain preservation may offer a dual benefit for weaned pigs by improving grain quality, reducing microbial load, and enhancing gut health and growth performance through better nutrient absorption and pathogen control [26].

This study aimed to evaluate the impact of varying dietary CP levels on the growth performance and intestinal health of weaned piglets. The experiment compared piglets offered conventional standard CP diets versus low CP diets, while also investigating the effects of conventional grain and OA-preserved grain. Several markers of growth and intestinal function were used to assess these impacts. The primary hypothesis was that reducing dietary CP levels in post-weaning diets would decrease the incidence of PWD, as indicated by lower faecal scores. Additionally, it was hypothesised that the inclusion of OA-preserved grain would improve protein digestion efficiency, thereby allowing for a reduction in dietary CP levels without compromising overall growth performance.

## 2. Materials and Methods

All experimental procedures conducted in the present study were approved by the University College Dublin Animal Research Ethics Committee (AREC-22-02-ODoherty) and were conducted in accordance with Irish legislation (SI no. 543/2012) and the EU directive 2010/63/EU for animal experimentation.

### 2.1. Grain Management and Quality

Winter wheat (cv. *JB Diego*) and spring barley (cv. *SY Errigal*), grains sourced from McAuley Feeds (Burtonstown, Co. Meath, Ireland), were utilised in this study and were subject to the same management and preservation practices outlined by Maher et al. [26]. The grain was grown and harvested during the 2021 growing season. The winter wheat was sown in October 2020, with the recommended practices of a three-spray fungicide programme and a three-split nitrogen application rate of 180 kg nitrogen/ha followed. The winter wheat was harvested in August 2021 in appropriate weather conditions, which resulted in a moisture content of 180 g/kg. The spring barley was sown in March 2021 and followed the recommended practices including a two-spray fungicide programme and a two-split nitrogen application rate of 140 kg/ha. The spring barley was harvested with a moisture content of 181 g/kg in August 2021. Prior to storage, both the wheat and barley were divided into two groups, with one group subject to drying using a continuous flow-type grain dryer (Cimbria, Thisted, Denmark) at 65 °C for 3 h before a 2 h cooling period. The moisture content of the wheat and barley after drying was 140 g/kg and 140.5 g/kg, respectively. The second grain group was preserved with a propionic acid mould inhibitor (OA), specifically a liquid surfactant (MycoCURB© ES Liquid; propionic acid (650 g/kg), ammonium propionate (70 g/kg), glycerol polyethyleneglycol ricinoleate (17.5 g/kg), and a carrier), sourced from Adesco Nutricines, Dungarvan, Co. Waterford, Ireland. The propionic acid mould inhibitor was applied at an inclusion level of 4 g/kg using spray action. To ensure an even distribution of the acid, a mixing auger was used. The grain was ventilated and stored before diet manufacture.

At the time of diet manufacture, 20 representative 500 g samples of the wheat and barley were collected using the grab sample technique and analysed for dry matter (DM), ash gross energy (GE), CP, crude fat, starch, mycotoxins and total mould count (TMC). The colony count technique (ISO21527-2:2008) was used to determine the TMC of the wheat and barley and was previously detailed by Laca et al. [27]. Liquid chromatography–mass spectrometry was used to determine the mycotoxin presence of aflatoxin B1, B2, fumonisin B1 and B2, G1 and G2, T-2 toxin, HT-toxin, deoxynivalenol (DON), ochratoxin A (OTA), and zearalenone (ZEN) as described by Soleimany et al. [28]. The chemical and mycotoxin analyses of the wheat and barley post-storage are presented in Table 1.

### 2.2. Experimental Design and Diets

This study was a 2 × 2 factorial design. Ninety-six newly weaned piglets (progeny of a Meatline Hermitage boar (Sion Road, Kilkenny, Ireland) × (Large White × Landrace sow)) were selected from a commercial farm with an average body weight of 7.4 kg (SD ± 0.82 kg). The piglets were blocked on live weight, sex, and litter of origin and assigned to one of four dietary stage 1 diets for the first 15 days of the experiment. The stage 1 diets comprised 478 g/kg of grain, with 328 g/kg being either dried or OA-preserved wheat and 150 g/kg being dried or OA-preserved barley. The rest of the composition (522 g/kg) consisted of either a standard protein concentrate or a low protein concentrate obtained from Cargill (Naas, Co., Kildare, Ireland), as outlined in Table 2. The stage 1 diets were as follows: (1) dried standard CP diet (20% CP); (2) OA-preserved standard CP diet (20% CP); (3) dried low CP diet (19% CP); and (4) OA-preserved low CP diet (19% CP). After 15 days, the piglets were offered a corresponding stage 2 diet for the remainder of the experimental period (D15–35). The diets comprised 553 g/kg of grain, with 403 g/kg being either dried or OA-preserved wheat and 150 g/kg being dried or OA-preserved barley. The rest of the composition (447 g/kg) consisted of either a standard CP concentrate or a low CP concentrate obtained from Cargill (Naas, Co., Kildare, Ireland), as outlined in Table 2. The stage 2 diets were as follows: (1) dried standard CP diet (19% CP); (2) OA-preserved standard CP diet (19% CP); (3) dried low CP diet (17% CP); and (4) OA-preserved low CP diet (17% CP). Celite (5 g/kg) was added to the stage 2 diets during manufacture to measure the coefficient of apparent total tract digestibility (CATTD) using the acid–insoluble ash (AIA) method described by McCarthy et al. [29]. The stage 1 diets were formulated to contain similar levels of standard ileal digestible lysine (13.0 g/kg) and net energy (11.0 MJ/kg). The stage 2 diets were also formulated to contain similar levels of standard ileal digestible lysine (12.0 g/kg) and net energy (10.8 MJ/kg). The levels of amino acids (AAs) were formulated to meet or exceed NRC requirements [8]. The standard and low CP diets were supplemented with synthetic lysine, methionine, threonine, tryptophan, and valine to meet amino acid requirements [8]. All diets were milled at the research facility and offered in meal form. The chemical and microbial analyses of the treatments are presented in Table 3.

### 2.3. Animal Management

The piglets were housed in groups of three in fully slatted pens (1.68 × 1.22 m) with 8 pens/treatment. The house temperature was thermostatically controlled at 30 °C for the first week and reduced by 2 °C per week thereafter. Humidity was maintained at 65%. Feed in the form of meal was provided ad libitum from two-space feeders alongside water from nipple drinkers. The piglets were weighed initially at the beginning of the experiments (day 0) and then every 7 days for the calculation of average daily gain (ADG). Offered feed was also weighed back every 7 days for the calculation of average daily feed intake (ADFI) and individual feed conversion ratio (FCR). Faecal scoring was conducted twice a day, every day by the same individual using a scale ranging from 1 to 5: 1 = hard, firm faeces; 2 = slightly soft faeces; 3 = soft, partially formed faeces; 4 = loose, semi-liquid faeces; and 5 = watery, mucous-like faeces, as previously described by Walsh et al. [30].

### 2.4. Sample Collection

On the 8th day post-weaning, one pig from each pen (n = 8) was humanely sacrificed for the collection of samples. The piglets received a lethal injection of pentobarbitone sodium (Euthanal solution, 200 mg/mL; Chanelle Pharma, Galway, Ireland) at a rate of 0.7 mL/kg body weight to the cranial vena cava. Euthanasia was completed by a trained individual in a separate room, out of sight and sound of the other piglets. The intact intestinal tract was promptly removed. Sections from the duodenum (10 cm from the stomach), the jejunum (60 cm from the stomach), and the ileum (15 cm from the caecum) were excised and fixed in 10% phosphate-buffered formalin, as previously detailed by Dowley et al. [31].

Tissue samples (1 cm) were dissected from the duodenum, jejunum, and ileum to establish relative gene expression of a range of functional categories, including nutrient transporters, cytokines, mucins, and pathogen recognition receptors using a quantitative real-time polymerase chain reaction (qPCR). The samples were dissected along the mesentery, emptied, and rinsed using sterile phosphate-buffered saline (Oxoid, Hampshire, UK). The tissue samples were stripped of the overlying smooth muscle before storage in 5 mL of RNAlater^®^ solution (Applied Biosystems, Foster City, CA, USA) overnight at 4 °C. The RNAlater^®^ was removed before storing the samples at −80 °C. Digesta from the ileum and colon was collected and stored in sterile containers (Sarstedt, Wexford, Ireland) on dry ice before storage at −80 °C for 16s rRNA sequencing and volatile fatty acid (VFA) analysis. Faecal samples were collected on day 30 of the experiment to determine the CATTD of nutrients. The CATTD was calculated using the internal marker AIA [29]. The following equation was utilised: CATTD of nutrient = (1 − [nutrient in faeces/nutrient in diet] × [AIA-diet/AIA-faeces]), where the nutrient concentrations in faeces and diet refer to the nutrient content (g/kg) in the DM of the faeces and diet, respectively. Similarly, AIA-diet and AIA-faeces represent the concentrations of acid–insoluble ash (AIA) in the dry matter of the diet and faeces [32].

### 2.5. Feed and Faecal Analysis

Representative samples were collected from the stage 1 and stage 2 diet of each dietary treatment at the time of diet formulation. Faecal samples were collected from every pen on day 30 PW and immediately frozen at −20 °C. The feed and faeces samples were then dried at 55 °C for 72 h to determine the DM content. The dried feed and faeces samples were then milled through a 1 mm screen (Christy and Norris Hammer Mill, Chelmsford, UK). Weighed samples were ignited at 550 °C for 6 h in a muffle furnace (Nabertherm, Bremen, Germany) to determine the crude ash content. The GE content of the feed and faeces was determined using an adiabatic bomb calorimeter (Parr Instruments, St Moline, IL, USA). Dietary crude fat levels were determined using light petroleum ether and Soxtec instrumentation (Tecator, Hillerod, Sweden). The nitrogen content of the diets and the faeces was determined using the Leco FP 528 instrument (Leco Instruments, Stockport, UK). A HPLC was utilised to assess the dietary amino acid concentrations as detailed by Iwaki et al. [33]. Dietary crude fibre content was determined according to the AOAC (1990 methodology (number 978.10)). The neutral detergent fibre (NDF) content of the feed was determined using the Ankom 220 Fibre Analyser (Ankom Technology, Macedon, NY, USA) in accordance with Van Soest et al. [34]. The chemical analysis of the dietary treatments are presented in Table 3.

### 2.6. Gut Morphological Analysis

The small intestine tissue was prepared for gut morphological analysis using standard paraffin embedding techniques as previously detailed by Rattigan et al. [35]. The tissue samples were cut at a thickness of 5 μm before being stained using haematoxylin and eosin. A light microscope with an image analyser (Image-Pro Plus; Media Cybernetics, Oxon, UK) was used to measure villus height (VH) and crypt depths (CD). The VH was determined by measuring from the tip of the villus down to the junction of the crypt and villus. The VH was measured from the base of the crypt to the junction of the crypt and villus. A minimum of 15 measurements of intact and well-orientated villi and crypt were taken from one duodenal, jejunal, and ileal tissue sample per pig.

### 2.7. Gene Expression in the Small Intestine

#### 2.7.1. RNA Extraction and cDNA Synthesis

Total RNA was extracted from 100 mg of tissue using TRIreagant (Sigma-Aldrich, St. Louis, MO, USA) according to the manufacturer’s instructions. The crude RNA was further purified using the GenElute Mammalian Total RNA miniprep kit (Sigma-Aldrich, St. Louis, MO, USA). A DNase step was incorporated using an on-column DNase 1 Digestion set (Thermo Scientific, Waltham, MA, USA). The quantity and purity of the total RNA were assessed by determining the ratio of the absorbance at 260 nm and 280 nm on a Nanodrop-ND1000 spectrophotometer (Thermo Scientific). Total RNA (2 µg) was reverse transcribed using a High-Capacity cDNA Reverse Transcription Kit (Applied Biosystems) and random primers in a final reaction volume of 40 µL. The cDNA was then made up to a volume of 400 µL using nuclease-free water.

#### 2.7.2. Quantitative Real-Time Polymerase Chain Reaction (qPCR)

The quantitative PCR (qPCR) reaction mix (20 µL) consisted of GoTaq qPCR Master Mic (10 µL) (Promega, Madison, WI, USA), forward and reverse primers (5 µM) (1.2 µL), nuclease-free water (3.8 µL), and cDNA (5 µL). All qPCR reactions were conducted in duplicate on the 7500 ABI Prism Sequence Detection System (Applied Biosystems, Foster City, CA, USA). The cycling conditions included a denaturation step of 95 °C for 10 min, followed by 40 cycles of 95 °C for 15 s and then 60 °C for 1 min. All the primers were designed using the Primer Express Software 5.0 (Applied Biosystems, Foster City, CA, USA) and synthesised by Eurofins (Milton Keynes, UK). Dissociation curves were created to verify the specificity of the subsequent PCR products.

The qPCR assay efficiencies were determined by plotting the cycling threshold (CT) values resulting from four-fold serial dilutions of cDNA against logs of their arbitrary quantities; only assays demonstrating 90–110% efficiency and single products were accepted in this analysis. Normalised relative quantities were determined using the software qbase PLUS 2.0 (Biogazelle, Ghent, Belgium) from stable reference genes *H3F3*, *YWAZ*, and *ACTB*. These reference genes were selected based on their M value (<1.5) generated by the GeNorm algorithm within GeNorm. The primer sequences utilised in the gene expression of the small intestine are presented in Table 4. These include *FABP2*, *SLC15A1*, *SLC2A1*, *CLDN1*, *TJP1*, *MUC2*, *IL1A*, *IL1B*, *IL6*, *CXCL8*, *IL17*, *IL22*, *TNF*, *FOXP3*, and *TLR4.*

### 2.8. Microbiological Analysis

#### 2.8.1. Microbial DNA Extraction

The microbial genomic DNA from the ileal and colonic digesta was extracted using QIAamp Powerfecal Pro DNA kit (Qiagen, West Sussex, UK) according to the manufacturer’s instructions. A Nanodrop ND-1000 Spectrophotometer (Thermo Scientific, Wilmington, DE, USA) was used to assess the quality and quantity of the resulting DNA.

#### 2.8.2. Illumina Sequencing

An Illuminia MiSeq platform was used to sequence the V3–V5 hypervariable region of the bacterial 16s rRNA gene in accordance with the service provider’s protocol (Eurofins Genomic, Eberberg, Germany). Universal primers containing adapter overhang nucleotide sequences for forward and reverse index primers were used to PCR-amplify the V3–V5 region. Amplicons were purified with the use of AMPure XP beads (Beckman Coulter, Indianapolis, IN, USA) and arranged for index PCR using Nextera XT index primers (Illumna, San Diego, CA, USA). The indexed samples were purified using the AMPure XP beads before quantification using a fragment analyser (Agilent, Santa Clara, CA, USA). Equal quantities from each were pooled and the Bioanalyser 7500 DNA kit (Agilent, Santa Clara, CA, USA) was used to quantify them before sequencing using the v3 chemistry (2 × 300 bp paired-end reads).

#### 2.8.3. Bioinformatics

Eurofins Genomics (Eberberg, Germany) completed the bioinformatic analysis using the Quantitative Insights into Microbial Ecology (Version 1.9.1) open source package [36]. Raw reads that passed the standard Illumina chastity filter were then demultiplexed in accordance with their index sequences (read quality > 30). The primer sequences were cut at the start of the raw forward and reverse reads. Primer sequences that did not match perfectly were removed to retain only high-quality reads. The paired-end reads were merged in order to form a single, longer read that covered the entire target region using the FLASH 2.200 software [37]. A minimum overlap size of 10 bp was required in order to reduce false-positive merges. Forward reads were only retained for subsequent assessment when merging was impossible. The merged reads were quality-filtered according to the expected and known length variations in the V3–V5 region. Forward reads that were retained were clipped at the end to a total length of 300 bp to eliminate low-quality bases. The retained and merged reads that contained ambiguous reads were removed. These filtered reads were used to generate the microbiome profile. The de novo algorithm of UCHIME [38] was used to detect and remove chimeric reads as implemented in the VSEARCH package [39]. The resulting collection of high-quality reads was processed using minimum entropy decomposition (MED), which sorted reads into operational taxonomic units (OTUs) [40]. DC-MEGABLAST alignments of the representative cluster sequences of the NCBI nucleotide sequence database were used for the taxonomic assignment of each OTU. A sequence identity of 70% across a minimum of 80% of the representative sequence was required to be considered a reference sequence. The bacterial taxonomic units were normalised using linear-specific copy numbers of appropriate marker genes to enhance estimates [41]. The data matrix comprised the normalised OTU table in combination with the phenotype metadata and phylogenetic tree. This was imported into the phyloseq package in R (Version 3.5.0). Differential abundance testing was performed on the tables from phyloseq at phylum, family, and genus levels.

The microbiome richness and diversity dynamics were computed using the observed, Fisher, Shannon, and Simpson indices. The diversity indices allocate different weights to various parameters of richness and evenness. Richness accounts for the differently observed taxa within a sample but does not take into account how frequently they are observed. The evenness of a sample compares the similarity of the population size of each species present within each individual sample [42,43].

Alpha diversity metrics were computed to determine the dynamics of richness and diversity in both ileal and colonic microbiomes. These included observed richness and the Fisher, Shannon, and Simpson indices. These metrics have differing emphases on richness and evenness. Richness measures the quantity of different taxa in each sample but does not measure how often they occur, while the evenness of a sample depicts the similarity between the population sizes of each species [42]. The observed alpha diversity measures the species richness as opposed to the Fisher, Shannon, and Simpson indices, which measure both richness and evenness [42,43]. The differentiation in the phylogenetic structure of OTUs between samples is assessed by beta diversity measurements. The data were normalised to enable the comparison of taxonomic feature counts across the different samples. The Bray–Curtis non-phylogenetic distance metric was then conducted using phyloseq in R [41,44].

### 2.9. Volatile Fatty Acid Analysis

The VFA concentrations of the colonic digesta were determined using gas–liquid chromatography as previously described by Clarke et al. [32]. One gram of digesta was diluted with distilled water (2.5 × sample weight) and centrifuged at 1400× *g* for 10 min using a Sorvall GLC-2B centrifuge (DuPont, Wilmington, DE, USA). One mL of the resulting supernatant and 1 mL of internal standard (0.05% 3-methyl-*n*-valeric acid in 0.15 M oxalic acid dihydrate) were mixed with 3 mL of distilled water and then centrifuged for 10 min at 500× *g*. The supernatant was filtered through a syringe filter (0.45 polytetrafluoroethylene (TFE)) into a chromatographic sample vial. An injection volume of 1 µL was injected into a Varian 3800 GC (Markham, ON, Canada) with an ECTM 1000 Grace column (15 m × 0.53 mm I.D) with a film thickness of 1.20 µm. The temperature programme was set to the range of 75–95 °C, increasing by 3 °C/min, and then to 95–200 °C, increasing by 20 °C/min, and this was held for 50 min. The detector and injector temperatures were 280 °C and 240 °C, respectively, while the total analysis time was 12.42 min.

### 2.10. Statistical Analysis

The growth parameters, faecal scores, small intestinal morphology, and gene expression data were checked for normality with the Shapiro–Wilk test in the UNIVARIATE procedure of SAS^®^ software version 9.4 (SAS Institute, Inc., Cary, NC, USA). The data were transformed when deemed necessary. The growth parameters (ADFI, ADG, FCR, and BW), as well as FS, were analysed by repeated measures using the PROC MIXED procedure. The data were divided into two time periods for the analysis: day 0–15 and day 15–35. The statistical model used included the grain preservation method, CP level, time of weighing, and their associated two- and three-way interactions. The initial weight was used as a covariate. For this experiment, the pen was the experimental unit.

The small intestinal morphology, VFA concentrations in digesta, gene expression data (Bonferroni adjusted *p* < 0.05), and bacterial alpha diversity were analysed using the SAS PROC GLM procedure. The statistical model incorporated grain preservation methods, CP levels, and their associated interactions. PROC GLIMMIX was used to generate least-square means with Benjamani–Hochberg adjusted *p*-values for nonparametric data associated with the microbial populations of ileal and colonic digesta. The results are presented as least-square means with their standard errors of the mean. When there was no interaction between the grain preservation method and dietary CP level, the results are presented as main effects. Significance was denoted as *p* < 0.05, with *p*-values between 0.05 ≤ *p* ≤ 0.10 defined as numerical tendencies.

## 3. Results

### 3.1. Grain Quality

The chemical and microbial analysis of the dried and OA-preserved wheat and barley at the time of diet manufacture is presented in Table 1. The OA-preserved wheat and barley had lower DM content compared to the dried wheat and barley. The OA-preserved barley had reduced levels of T-2 toxin and HT-2 toxin compared to the dried barley. The OA-preserved wheat and barley had reduced levels of OTA compared to the dried wheat and barley.

### 3.2. Growth Performance and Faecal Scores

The effects of the grain preservation method and dietary CP levels during the PW period (d 0–15, d 15–35) are presented in Table 5.

There were interactions between the grain preservation method, CP levels, and time on the ADG and FCR (*p* < 0.05); during the first period (d 0–15), the OA-preserved grain diet with standard CP increased ADG compared to the dried grain diet with standard CP (396 vs. 325 g/day, SEM 22.10). There was no difference in ADG between the OA-preserved grain diet with low CP and the dried grain diet with low CP (366 vs. 360 g/day, SEM 22.10). During the second period (d 15–35), the OA-preserved grain diet with low CP increased ADG compared to the dried grain diet with low CP (630 vs. 560 g/day, SEM 22.10); however, there was no difference in ADG between the OA-preserved diet with standard CP and the dried grain diet with standard CP (649 vs. 657 g/day, SEM 22.10). During the first period (d 0–15), the OA-preserved grain diet with standard CP improved the FCR compared to the dried grain diet with standard CP (1.04 vs. 1.29, SEM 0.052). There was no difference in FCRs between the OA-preserved grain diet with low CP and the dried grain diet with low CP (1.12 vs. 1.20, SEM 0.052). During the second period (d 15–35), there was no effect of grain preservation on the FCR in the standard CP and the low CP diets.

Piglets offered OA-preserved grain had a higher final BW compared to those offered dried grain (24.5 kg vs. 23.1 kg, SEM 0.256; *p* < 0.05). There was no grain x CP interaction on FS. During the first 15 days, piglets offered low CP diets had reduced FS compared to piglets offered standard CP diets (2.24 vs. 2.19 SEM 0.019; *p* < 0.05).

### 3.3. Coefficient of Apparent Total Tract Digestibility

The effects of the grain preservation method and dietary CP on the CATTD of nutrients on day 30 post-weaning are presented in Table 6. There were grain × CP interactions on the CATTD of DM, OM, N, and GE (*p* < 0.05); piglets offered the OA-preserved grain with low CP had increased CATTD of DM, OM, and GE compared to those offered the OA-preserved grain with standard CP, but there was no difference in the CATTD of DM, OM, and GE between the dried grain with standard CP and the dried grain with low CP. Piglets offered the dried grain with low CP had reduced CATTD of N compared to those offered the dried grain with standard CP; however, there was no difference in the CATTD of N between the OA-preserved grain with low CP and the OA-preserved grain with standard CP. Piglets offered OA-preserved grain had increased CATTD of ash compared to those offered dried grain (60.09 vs. 58.59, SEM 0.460; *p* < 0.05).

### 3.4. Small Intestinal Morphology

The effects of the grain preservation method and dietary CP levels on small intestinal morphology are presented in Table 7. There was no effect of the grain preservation method, dietary CP level, or grain × CP interaction on duodenal, jejunal, or ileal VH, CD, or VH:CD ratios.

### 3.5. Gene Expression Analysis

The effects of the grain preservation method and dietary CP level on the relative expression of genes that were differentially expressed in the small intestine are presented in Table 8.

In the duodenum, piglets offered OA-preserved grain had reduced expression of *IL1A* relative to those offered dried grain (0.89 vs. 1.35, SEM 0.152; *p* < 0.05). Piglets offered low CP diets had increased expression of *IL1B* compared to piglets offered standard CP diets (1.80 vs. 0.95, SEM 0.287; *p* < 0.05). In the jejunum, piglets offered OA-preserved grain had reduced expression of *IL17* compared to those offered dried grain (0.88 vs. 2.13, SEM 0.405; *p* < 0.05). While, in the ileum, piglets offered OA-preserved grain had reduced expression of *IL17* compared to those offered dried grain (0.82 vs. 1.45, SEM 0.167; *p* < 0.05). There was no interaction between the grain preservation method and dietary CP on the expression of genes in the duodenum, jejunum, or ileum.

### 3.6. Differential Bacterial Abundance Analysis

#### 3.6.1. Bacterial Richness and Diversity

There was no effect of the grain preservation method or dietary CP levels on the observed, Fisher, Shannon, or Simpson index diversity measures in the digesta collected from the ileum and colon (*p* > 0.05). Similarly, there were no differences in beta diversity in the ileal and colonic microbiome based on visualisation using the Bray–Curtis distance matrix and multi-dimensional scaling.

#### 3.6.2. Differently Abundant Phlya

The effects of the grain preservation method and dietary CP level on the relative abundance of bacterial phyla are presented in Table 9.

In the ileum, the predominant phyla were Firmicutes (~94.50%). There was no effect of the grain preservation method, CP level, or grain preservation × CP interaction on the relative abundance of bacterial phlya in the ileum (*p* > 0.05).

In the colon, the predominant phyla were Firmicutes (~76.22%), Bacteroidetes (~11.50%), Actinobacteria (~3.91%), Tenericutes (~0.72%), and Spirochaetes (~0.61%). There was an interaction between the grain preservation method and CP levels on the relative abundance of Tenericutes; piglets offered the OA-preserved grain with low CP had an increased relative abundance of Tenericutes compared to the piglets offered the OA-preserved grain with standard CP, but there was no difference in the relative abundance of Tenericutes between the piglets offered dried grain with standard CP and those offered the dried grain with low CP (*p* = 0.05). Piglets offered OA-preserved grain had an increased relative abundance of Bacteroidetes compared to those offered dried grain (15.00 vs. 8.00, SEM 0.968; *p* < 0.001). The low CP diets resulted in an increased relative abundance of Bacteroidetes (13.08 vs. 9.91, SEM 0.904) and Spirochaetes (1.04 vs. 0.19, SEM 0.255) compared to the standard CP diets (*p* < 0.05).

#### 3.6.3. Differently Abundant Families

The effects of the grain preservation method and dietary CP level on the relative abundance of bacterial families are presented in Table 10.

In the ileum, there were interactions between the grain preservation method and dietary CP levels on the relative abundance of *Clostridiaceae* and *Streptococcaceae* (*p* < 0.01); The OA-preserved grain diet with standard CP reduced the relative abundance of *Clostridiaceae* compared to the dried grain diet with standard CP, but there was no effect of the grain preservation method on *Clostridiaceae* in the low CP diets. The OA-preserved grain diet with standard CP reduced the relative abundance of *Streptococcaceae* compared to the dried grain diet with standard CP; however, the OA-preserved grain diet with low CP increased *Streptococcaceae* compared to the dried grain diet with low CP. Piglets offered OA-preserved grain had an increased relative abundance of *Lactobacillaceae* compared to those offered dried grain (82.38 vs. 67.05, SEM 2.76; *p* < 0.001). Piglets offered low CP diets had a reduced relative abundance of *Lactobacillaceae* compared to those offered the standard CP diets (70.61 vs. 78.82, SEM 2.66; *p* < 0.05).

In the colon, there were interactions between the grain preservation method and dietary CP levels on the relative abundance of *Lachnospiraceae*, *Propionibacteriaceae*, *Spiroplasmataceae*, *Rikenellaceae*, and *Christensenellaceae* (*p* < 0.05). Piglets offered the OA-preserved grain diet with low CP had a reduced relative abundance of *Lachnospiraceae* and *Christensenellaceae* compared to those offered the dried grain diet with low CP; however, there was no effect of the grain preservation method on the abundance of *Lachnospiraceae* and *Christensenellaceae* in the standard CP diets. Piglets offered the OA-preserved grain diet with low CP had an increased relative abundance of *Spiroplasmataceae* and *Rikenellaceae* compared to those offered the dried grain diet with low CP; however, there was no effect of the grain preservation method on the abundance of *Spiroplasmataceae* and *Rikenellaceae* in the standard CP diets. Piglets offered the dried grain diet with low CP had an increased relative abundance of *Propionibacteriaceae* compared to those offered the dried grain diet with standard CP, but there was no difference in the abundance of *Propionibacteriaceae* between the OA-preserved grain diet with low CP and the OA-preserved grain diet with standard CP.

Piglets offered OA-preserved grain had a reduced relative abundance of *Lactobacillaceae* compared to piglets offered dried grain (9.07 vs. 14.21, SEM 0.962; *p* < 0.01). Piglets offered OA-preserved grain had an increased relative abundance of *Eubacteriacea* (3.30 vs. 2.15 SEM 0.454), *Veillonellaceae* (0.70 vs. 0.14, SEM 0.218), and *Prevotellaceae* (12.52 vs. 6.52, SEM 0.884) compared to those offered dried grain (*p* < 0.05). Piglets offered the low CP diets had a reduced relative abundance of *Lactobacillaceae* compared to those offered the standard CP diets (7.70 vs. 15.58, SEM 1.012; *p* < 0.001). Piglets offered the low CP diets had an increased relative abundance of *Eubacteriaceae* (3.68 vs. 1.77, SEM 0.480) and *Spirochaetaceae* (1.08 vs. 0.20, SEM 0.260) compared to those offered the standard CP diets (*p* < 0.05).

#### 3.6.4. Differently Abundant Genera

The effects of the grain preservation method and dietary CP level on the relative abundance of bacterial genera are presented in Table 11.

In the ileum, there was an interaction between the grain preservation method and CP level on the relative abundance of *Clostridium* and *Streptococcus* (*p* < 0.05); piglets offered low CP diets had a reduced relative abundance of *Clostridium* in the dried grain diet condition; however, the lowering of protein had no effect on the relative abundance of *Clostridium* in the OA-preserved grain diet condition. Piglets offered the OA-preserved grain diet with low CP had an increased relative abundance of Streptococcus compared to those offered the OA-preserved grain diet with standard CP, but there was no difference in the abundance of Streptococcus between piglets offered the dried grain diet with low CP and the dried grain diet with standard CP. Piglets offered OA-preserved grain had an increased relative abundance of *Lactobacillus* compared to those offered dried grain (82.72 vs. 68.29, SEM 2.761; *p* < 0.001).

In the colon, there were significant interactions between the grain preservation method and CP levels on the relative abundance of *Faecalibacterium*, *Clostridium*, *Spiroplasma*, *Anaerocella*, *Dorea*, *Prevotella*, and *Christensenella* (*p* < 0.05); piglets offered the OA-preserved grain diet with standard CP had an increased relative abundance of *Faecalibacterium* and *Prevotella* compared to those offered the dried grain diet with standard protein; however, there was no effect of the grain preservation method on *Faecalibacterium* and *Prevotella* in the low CP diets. Piglets offered the OA-preserved grain diet with low CP had increased relative abundance of *Clostridium* and *Spiroplasma* compared to those offered dried grain with low CP, but there was no effect of the grain preservation method on *Clostridium* and *Spiroplasma* in the standard CP diets. Piglets offered the OA-preserved grain diet with low CP had an increased relative abundance of *Anareocella* compared to those offered the OA-preserved grain diet with standard CP; however, there was no difference in the abundance of *Anaerocella* between the dried grain diet with low CP and the dried grain diet with standard CP. Piglets offered the dried grain diet with low CP had an increased relative abundance of *Dorea* compared to those offered the dried grain diet with standard CP; however, there was no difference in the abundance of *Dorea* between the OA-preserved grain diet with low CP and the OA-preserved grain diet with standard CP.

Piglets offered OA-preserved grain had increased relative abundance of *Roseburia* compared to those offered dried grain (3.38 vs. 1.68, SEM 0.479; *p* < 0.001). Piglets offered OA-preserved grain had reduced abundance of *Lactobacillus* (9.12 vs. 14.48, SEM 0.971), *Collinsella* (0.18 vs. 1.21, SEM 0.279), *Gemmiger* (5.54 vs. 7.98, SEM 0.706), *Ruminococcus* (0.99 vs. 2.28, SEM 0.377), *Blautia* (1.07 vs. 2.72, SEM 0.413) and *Pseudoflavonifractor* (0.61 vs. 1.51, SEM 0.316) compared to those offered dried grain (*p* < 0.005). Piglets offered low CP diets had increased relative abundance of *Eubacterium* (3.31 vs. 1.77, SEM 0.470) and *Treponema* (0.97 vs. 0.14, SEM 0.246) compared to those offered standard CP diets (*p* < 0.005). Piglets offered low CP diets had reduced relative abundance of *Lactobacillus* compared to those offered standard CP diets (7.88 vs. 15.71, SEM 1.016; *p* < 0.005).

### 3.7. Volatile Fatty Analysis

The effects of the grain preservation method and dietary CP levels on the molar proportions and total concentrations of VFAs in the colon are presented in Table 12. There was an interaction between the grain preservation method and CP level on the total VFA concentration (*p* < 0.05); piglets offered the dried grain diet with low CP had reduced total VFA concentrations compared to those offered the dried grain diet with standard CP; however, there was no difference in total VFA concentrations between piglets offered the OA-preserved grain diet with low CP and the OA-preserved grain diet with standard CP. Piglets offered low CP diets had increased molar propionate (0.336 vs. 0.281, SEM 0.0056) and butyrate proportions (0.187 vs. 0.153, SEM 0.0085) compared to those offered standard CP diets (*p* < 0.01). Piglets offered low CP diets had reduced molar acetate proportions compared to those offered standard CP diets (0.423 vs. 0.506, SEM 0.0114; *p* < 0.001).

## 4. Discussion

This study examined the effects of low CP diets on growth performance and gut health in post-weaned piglets, and whether OA-preserved grain could mitigate the negative impacts of CP reduction. The results show that low CP diets consistently reduced FS and enhanced colonic butyrate levels compared to standard CP diets, suggesting improved intestinal function. During the first 15 days post-weaning, piglets offered OA-preserved grain with standard CP exhibited better FCRs than those offered dried grain with the same CP level. However, in low CP diets, OA-preserved grain did not provide additional FCR benefits. From days 15 to 35, piglets offered dried grain with low CP showed reduced ADG compared to those on dried grain with standard CP. In contrast, piglets offered OA-preserved grain with low CP maintained ADG comparable to those on OA-preserved grain with standard CP. By the end of the study, OA-preserved grain led to higher final BW compared to dried grain, indicating its potential to support growth and intestinal health in low CP diets.

Pigs are generally offered high CP diets post-weaning to maximise growth and FCRs [45]. However, their immature digestive systems are susceptible to colonic protein fermentation processes, which can worsen post-weaning diarrhoea (PWD) [46,47]. Reducing dietary CP has been proposed to limit the undigested protein available for fermentation and mitigate PWD [48]. Although no PWD was observed in this study, low CP diets reduced FS, consistent with previous research [49,50,51]. This improvement may be linked to microbial shifts induced by low CP diets. Given the relationship between gut microbiota and PWD [52], it is likely that increased populations of beneficial bacteria contributed to the improved FS observed in this study. This effect may be more pronounced in commercial swine production settings rather than in research environments, due to increased bacterial load and social stress.

Dietary CP levels are known to influence gut microbiome composition. High CP diets increase the abundance of proteolytic bacteria such as *Clostridium*, *Propionibacterium*, and *Streptococcus*, while reducing populations of beneficial bacteria like *Eubacterium*, *Ruminococcus*, *Butyrivibrio*, and *Blautia* [53,54]. In this study, all dietary treatments had reduced ileal *Clostridium* compared to the dried grain diet with standard CP, but, surprisingly, the OA-preserved grain diet with low CP had the highest relative abundance of ileal *Streptococcus* of all treatments. Although some bacteria belonging to the genera *Streptococcus* [55] are health-promoting, others can be detrimental for post-weaned pig health [56]. However, considering the overall positive effects of the OA-preserved grain diet with low CP on growth performance, it could be assumed that the increased abundance of ileal *Streptococcus* in the treatment was not pathogenic in nature. Additionally, the low CP diets increased the abundance of *Eubacterium* and *Faecalibacterium*, particularly *Faecalibacterium prausnitzii*, a key butyrate producer known for its anti-inflammatory influence and role in maintaining gut barrier integrity [57,58]. Consistent with this microbial profile, low CP diets enhanced colonic butyrate concentrations. Short-chain fatty acids, including butyrate, are essential for nutrient absorption, anti-inflammatory responses, and energy production, meeting 10–13% of a pig’s energy needs [59,60]. Butyrate supports intestinal development, inhibits enteric pathogens [61], reduces pro-inflammatory cytokines [62], and promotes regulatory T cell (Treg) activation [63]. Despite these benefits, low CP diets increased duodenal expression of the pro-inflammatory cytokine *IL1B*, consistent with a similar study by Rattigan et al. [64]. Weaning is often associated with increased pro-inflammatory cytokine expression, compromising intestinal integrity and contributing to PWD [65]. The replacement of dietary CP with synthetic amino acids in mice has been shown to reduce regulatory T cell (Treg) production and exacerbate inflammation, due to the inability of synthetic amino acids to bind to immune cells [66]. These findings suggest that while low CP diets modulate the microbiome and improve intestinal health, amino acid supplementation may exacerbate inflammation, contributing to the inconsistent growth responses observed in low CP diets.

While low CP diets often reduce growth performance due to challenges in meeting amino acid requirements [67,68], OA supplementation may help address these issues. The inclusion of OAs may present a viable solution to overcome the challenges associated with amino acid supplementation. Organic acids have been shown to improve protein utilisation, reduce inflammation, and enhance growth performance [69,70,71]. In this study, OA-preserved grain had reduced total mycotoxin contamination compared to dried grain, while OA-preserved barley had lower HT-2 toxin levels compared to dried barley. Mycotoxins are harmful fungal metabolites that can contaminate grain following harvest or during storage [72]. Specifically, HT-2 toxin stimulates the production of pro-inflammatory cytokines while suppressing anti-inflammatory cytokines [73]. Mycotoxin exposure can alter the intestinal microbiome [74] and reduce feed intake [72]. Therefore, the reduced mycotoxin burden in OA-preserved grain may have contributed to the improved growth performance and microbial changes observed in piglets fed preserved grain.

Piglets fed OA-preserved grain with standard CP achieved the best FCR during the first 15 days. On day 8 post-weaning, this group exhibited higher relative abundances of beneficial microbes, including *Lactobacillus* in the ileum and *Faecalibacterium*, *Roseburia*, and *Prevotella* in the colon. These microbes play key roles in gut health. *Lactobacillus* promotes antimicrobial peptide production, mucus secretion, and tight junction function [75]. *Prevotella* and *Lactobacillus* metabolise plant polysaccharides, aiding dietary adaptation and increasing SCFA production [76,77]. *Roseburia* and *Faecalibacterium* are butyrate producers with anti-inflammatory properties, supporting immune regulation [57,78]. All these bacteria are associated with improved FCRs [79,80,81]. The increased abundance of these microbes in piglets offered by OA-preserved grain likely explains the reduced pro-inflammatory cytokines and enhanced growth performance. Piglets offered OA-preserved grain had reduced duodenal *IL1A* and jejunal and ileal *IL17* expression compared to dried grain. The upregulation of *IL1A* and *IL17* can drive inflammation in the gut [82,83], which in turn diverts nutrients away from growth and towards the immune system [84]. These effects likely contributed to the improved FCRs observed during the first 15 days in piglets fed OA-preserved grain.

From days 15 to 35, OA-preserved grain effectively supported low CP diets. While dried grain with low CP reduced ADG and colonic SCFA concentrations, the inclusion of OA-preserved grain in low CP diets maintained ADG and SCFA levels similar to those seen in pigs fed standard CP diets. This improvement in growth may be attributed to the ability of OA-preserved grain to maintain the CATTD of nitrogen when dietary CP levels were lowered. Low CP diets are typically associated with reduced growth performance due to issues meeting amino acid requirements [67,68]. In this study, although the NRC guidelines [8] were met, the levels of branched-chain amino acids (BCAAs) and non-essential amino acids (NEAAs) were lower in the low-CP diets compared to the standard CP diets. This reduction in amino acid levels likely contributed to the decreased performance observed during this period. While NEAAs have traditionally been considered non-essential, they are now recognised as conditionally essential for the growth and development of weaned pigs [85]. The inclusion of OAs appears to be a promising solution to the challenges posed by amino acid supplementation in low-CP diets. In support of this, Maher et al. [26] demonstrated that post-weaned pigs consuming OA-preserved grain showed higher ileal N digestibility. This suggests that OA-preserved grain improves N efficiency, allowing for reduced dietary CP levels without compromising growth. Furthermore, the decrease in SCFA production in the dried grain, low CP diet aligns with findings from Luise et al. [86], who observed a link between lower dietary CP and reduced SCFA production. Given that SCFAs serve as essential energy sources [87], maintaining their production likely played a role in preserving growth performance in the OA-preserved grain group.

## 5. Conclusions

Organic acid-preserved low crude protein diets effectively improved intestinal health and maintained growth performance post-weaning. Low crude protein diets reduced faecal scores, promoted beneficial microbial shifts, and enhanced colonic butyrate levels but increased duodenal *IL1B* expression. Organic acid-preserved grain enhanced performance by reducing intestinal inflammation and stimulating beneficial microbes, while organic acid-preserved grain also maintained nitrogen digestibility when CP levels were lowered. These findings highlight the potential of organic acid-preserved low crude protein diets to support piglet growth and gut health. These findings suggest that organic acid-preserved low crude protein diets are a promising strategy to support piglet growth and gut health post-weaning. However, further studies are recommended to validate these benefits in more challenging, stress-inducing environments.

## Figures and Tables

**Table 1:** The chemical and microbiological analysis of experimental grain after storage (g/kg) unless otherwise stated.

Cereal Crop Type	Wheat		Barley	
Grain Preservation Method	Dried	OA-Preserved	Dried	OA-Preserved
Analysis post storage (g/kg)				
DM	873.5	840.5	873.5	848.5
Ash	16.2	15.8	19.5	19.0
GE (MJ/kg)	15.9	15.2	16.1	15.6
Crude protein	89.0	84.5	103.5	87.5
Crude fibre	25.5	23.5	57.5	52.0
Starch	626.5	608.5	530.0	504.0
Fat	14.5	14.0	15.5	14.0
TMC (cfu/g)	37,000	3800	27,000	2400
Mycotoxin levels (μg/kg) ^a^				
Deoxynivalenol	<75	<75	<75	<75
T-2 toxin	<4.00	<4.00	6.96	<4.00
HT-2 toxin	<4.00	<4.00	30.1	8.66
Zearalenone	<10	<10	<10	<10
Ochratoxin A	3.2	<1.00	1.8	<1.0

Abbreviations: DM, dry matter; GE, gross energy; TMC, total mould count. ^a^ The following mycotoxins were below detectable levels: aflatoxin B1, B2, G1, and G2.

**Table 2:** Ingredients and chemical composition of experimental stage 1 and stage 2 diets (g/kg unless otherwise stated).

	Dietary Treatments *							
	Stage 1 Diets				Stage 2 Diets			
Grain Preservation Method	Dried	OA-Preserved	Dried	OA-Preserved	Dried	OA-Preserved	Dried	OA-Preserved
Crude Protein Level	Standard	Standard	Low	Low	Standard	Standard	Low	Low
Ingredients (g/kg)								
Wheat	328	328	328	328	403	403	403	403
Barley	150	150	150	150	150	150	150	150
Maize	95	95	170	170	81	81	145	145
Full fat soya	170	170	140	140	145	145	119	119
Soya bean meal	95	95	70	70	82	81	60	60
Soya bean concentrate	40	40	60	60	34	34	51	51
Whey powder	50	50	50	50	43	43	43	43
Soya oil	30	30	30	30	26	26	26	26
Starch	5	5	-	-	5	5	-	-
Salt	2	2	2	2	2	2	2	2
Mono calcium Phosphate	4.2	4.2	4.2	4.2	4.2	4.2	4.2	4.2
Calcium carbonate	4.5	4.5	4.5	4.5	4.5	4.5	4.5	4.5
Lysine HCl	2.5	2.5	4.9	4.9	2.5	2.5	4.9	4.9
DL-Methionine	2	2	2.5	2.5	2	2	2.5	2.5
L-Threonine	1.8	1.8	2.7	2.7	1.8	1.8	2.7	2.7
Tryptophan	0.3	0.3	0.7	0.7	0.3	0.3	0.7	0.7
Valine	-	-	0.5	0.5	-	-	0.5	0.5

* Treatments: stage 1 diets were offered for the first 15 days of the experiment: (1) dried standard CP (20% CP); OA-preserved standard CP (20% CP); (3) dried low CP (19% CP); and (4) OA-preserved low CP (19% CP). After 15 days, piglets were offered a corresponding stage 2 diet for the remainder of the experiment: (1) dried standard CP (19% CP); (2) OA-preserved standard CP (19% CP); (3) dried low CP (17% CP); and (4) OA-Preserved Low CP (17% CP).

**Table 3:** The analysed composition of experimental stage 1 and stage 2 diets (g/kg unless otherwise stated).

	Dietary Treatments *							
	Stage 1 Diets				Stage 2 Diets			
Grain Preservation Method	Dried	OA-Preserved	Dried	OA-Preserved	Dried	OA-Preserved	Dried	OA-Preserved
Crude Protein Level	Standard	Standard	Low	Low	Standard	Standard	Low	Low
DM	895.0	886.0	896.0	882.5	894.0	878.5	892.5	888.0
Ash	45.5	44.5	39.5	36.5	43.0	39.5	33.5	32.0
GE (MJ/kg)	17.1	16.7	16.7	16.7	16.9	16.6	16.7	16.5
Crude fat	63.0	61.0	58.5	57.0	57.0	56.0	54.0	53.0
Crude protein	197.5	191.5	185.0	182.5	188.5	187.5	172.5	175.0
Crude fibre	28.5	25.5	25.0	23.5	28.5	23.5	25.5	22.0
NDF	111.5	100.5	107.0	98.0	112.5	98.0	106.5	95.0
ADF	33.5	28.5	31.0	28.5	34.0	28.5	30.5	27.0
Starch	319.0	299.0	354.0	350.0	340.0	336.5	383.5	375.5
Amino Acids								
Lysine	15.67	15.65	15.57	15.55	14.07	14.06	14.24	14.25
Threonine	11.01	11.04	10.71	10.70	10.98	10.10	9.99	9.96
Methionine and cysteine	10.29	10.31	10.03	10.01	9.67	9.70	9.55	9.53
Leucine	19.37	19.35	17.49	17.45	14.04	14.06	14.27	14.30
Iso-Leucine	10.88	10.86	9.53	9.56	9.87	9.84	8.72	8.76
Arginine	14.34	14.37	12.05	12.06	13.05	13.08	11.10	11.07
Histidine	5.92	5.95	5.18	5.20	5.45	5.44	4.79	4.78
Phenylalanine	11.24	11.27	9.73	9.76	10.30	10.33	9.02	9.04
Tyrosine	7.51	7.53	6.56	6.54	6.87	6.90	6.09	6.07
Alanine	10.37	10.35	9.33	9.36	9.40	9.44	8.53	8.55
Aspartic	22.48	22.45	19.75	19.78	19.97	19.95	17.74	17.77
Glutaminc	46.99	47.1	41.92	41.90	44.2	44.6	40.05	40.01
Glycine	9.00	9.03	7.81	7.78	8.35	8.36	7.33	7.30
Serine	11.41	11.40	9.93	9.96	10.51	10.50	9.23	9.23
Proline	15.63	15.60	14.32	14.34	14.88	14.86	13.75	13.78
Tryptophan	2.72	2.74	2.73	2.74	2.56	2.55	2.62	2.60
Valine	12.02	12.04	10.62	10.66	8.62	8.63	7.83	7.80
TMC (cfu/g)	6200	3700	4800	3300	4300	3700	5600	4000
Mycotoxin levels (mg/kg) ^a^								
Deoxynivalenol	<75	<75	<75	<75	<75	<75	<75	<75
T-2 toxin	5.62	<4.00	<4.00	<4.00	<4.00	<4.00	<4.00	<4.00
HT-2 toxin	23.1	13.3	14.1	11.3	<15.7	<10.8	<10.7	<10.6
Zearalenone	35	37	30	27	39	31	23	25
Ochratoxin	1.09	<1.0	2.39	<1.00	<1.65	<1.00	<1.60	<1.00

* Treatments: stage 1 diets were offered for the first 15 days of the experiment: (1) dried standard CP (20% CP); (2) OA-preserved standard CP (20% CP); (3) dried low CP (19% CP); and (4) OA-preserved low CP (19% CP). After 15 days, piglets were offered a corresponding stage 2 diet for the remainder of the experiment: (1) dried standard CP (19% CP); (2) OA-preserved standard CP (19% CP); (3) dried low CP (17% CP); and (4) OA-preserved low CP (17% CP). Abbreviations: DM, dry matter; GE, gross energy; NDF, neutral detergent fibre; ADF, acid detergent fibre. ^a^ The following mycotoxins were below the listed detectable levels: aflatoxin B1, B2, G1, and G2 (<1 μg/kg); fumonisin B1 (<125 μg/kg) and fumonisin B2 (<50 μg/kg).

**Table 4:** Panel of primer sequences for qPCR analysis.

Target Gene	Gene Name	Accession no.	Forward Primer (5′-3′) Reverse Primer (5′-3′)
Nutrient transporters			
*FABP2*	Fatty Acid Binding Protein 2	NM_001031780.1	F: CAGCCTCGCAGACGGAACTGAA R: GTGTTCTGGGCTGTGCTCCAAGA
*SLC2A1*	Solute Carrier Family 2 Member 1	XM_003482115.1	F: TGCTCATCAACCGCAATGA R: GTTCCGCGCAGCTTCTTC
*SLC15A1*	Solute Carrier Family 15 Member 1	NM_214347.1	F: GGATAGCCTGTACCCCAAGCT R: CATCCTCCACGTGCTTCTTGA
Inflammatory markers			
*IL1A*	Interleukin 1A	NM_214029.1	F: CAGCCAACGGGAAGATTCTG R: ATGGCTTCCAGGTCGTCAT
*IL1B*	Interleukin 1B	NM_001005149.1	F: TTGAATTCGAGTCTGCCCTGT R: CCCAGGAAGACGGGCTTT
*IL6*	Interleukin 6	NM_214399.1	F: GACAAAGCCACCACCCCTAA R:CTCGTTCTGTGACTGCAGCTTATC
*CXCL8*	C-X-C Motif Chemokine Ligand 8	NM_213867.1	F: TGCACTTACTCTTGCCAGAACTG R: CAAACTGGCTGTTGCCTTCTT
*IL10*	Interleukin 10	NM_214041.1	F: GCCTTCGGCCCAGTGAA R: AGAGACCCGGTCAGCAACAA
*IL17*	Interleukin 17	NM_001005729.1	F: CCCTGTCACTGCTGCTTCTG R: TCATGATTCCCGCCTTCAC
*IL22*	Interleukin 22	XM_001926156.1	F: GATGAGAGAGCGCTGCTACCTGG R: GAAGGACGCCACCTCCTGCATGT
*TNF*	Tumour Necrosis Factor	NM_214022.1	F: TGGCCCCTTGAGCATCA R: CGGGCTTATCTGAGGTTTGAGA
*FOXP3*	Forkhead Box P3	NM_001128438.1	F: GTGGTGCAGTCTCTGGAACAAC R: AGGTGGGCCTGCATAGCA
Tight junctions			
*TJP1*	Tight Junction Protein 1	XM_021098827.1	F: TGAGAGCCAACCATGTCTTGAA R: CTCAGACCCGGCTCTCTGTCT
*CLDN1*	Claudin 1	NM_001244539.1	F: CTGGGAGGTGCCCTACTTTG R: TGGATAGGGCCTTGGTGTTG
Toll like receptors			
*TLR4*	Toll-Like Receptor 4	NM_001293317.1	F: TGCATGGAGCTGAATTTCTACAA R: GATAAATCCAGCACCTGCAGTTC
Mucins			
*MUC2*	Mucin 2	AK231524	F: CAACGGCCTCTCCTTCTCTGT R: GCCACACTGGCCCTTTGT
Reference genes			
*H3F3A*	Histone H3.3	NM_213930.1	F: CATGGCTCGTACAAAGCAGA R: ACCAGGCCTGTAACGATGAG
*YWHAZ*	Tyrosine 3-Monooxygenase/Tyrtophan 5-Monooxygenase Activation Protein Zeta	NM_001315726.1	F: GGACATCGGATACCCAAGGA R: AAGTTGGAAGGCCGGTTAATTT
*ACTB*	Actin Beta	XM_001927228.1	F:GGACATCGGATACCCAAGGA R:AAGTTGGAAGGCCGGTTAATTT

**Table 5:** The effect of dietary treatment on pig growth performance and faecal scores (least-square means with their standard errors).

	Treatments *									*p*-Values			
	Dried Standard CP		OA-Preserved Standard CP		Dried Low CP		OA-Preserved Low CP		SEM	Grain	Protein	Grain × Protein	Time × Grain × Protein
	D0–15	D15–35	D0–15	D15–35	D0–15	D15–35	D0–15	D15–35					
ADFI (g/DM/day)	397	853	401	877	413	780	403	844	19.32	0.214	0.196	0.700	0.162
ADG (g/d)	325	657	396	649	362	560	366	630	22.10	0.055	0.127	0.888	**0.009**
FCR **	1.29	1.30	1.04	1.38	1.20	1.42	1.12	1.36	0.053	0.049	0.591	0.841	**0.032**
BW (kg)	12.27	23.59	13.13	24.83	12.82	22.65	12.90	24.23	0.538	**0.048**	0.513	0.812	0.304
FS	2.24	-	2.23	-	2.18	-	2.19	-	0.027	0.967	**0.050**	0.582	0.929

Treatments: BW, body weight; ADG, average daily gain; ADFI, average daily feed intake; FCR, feed conversion ratio; FS, faecal score. * A total of eight replicates were used per treatment group (replicate = pen, 3 pigs/pen) ** FCR calculated on a dry matter basis (kg/kg DM).

**Table 6:** The effect of dietary treatment on the coefficient of apparent total tract digestibility of dry matter (DM), organic matter (OM), ash, nitrogen (N), and gross energy (GE) on day 30 post-weaning (least-square means with their SEM).

	Treatment *					*p* Values		
Grain Preservation Method	Dried	OA-Preserved	Dried	OA-Preserved	SEM	Grain	Protein	Grain × Protein
Dietary Crude Protein Level	Standard	Standard	Low	Low				
DM	0.851 ^ab^	0.843 ^a^	0.845 ^ab^	0.853 ^b^	0.0035	0.953	0.464	**0.023**
OM	86.81 ^ab^	85.97 ^a^	86.30 ^ab^	87.08 ^b^	0.3409	0.920	0.371	**0.019**
Ash	59.47	60.00	57.70	60.18	0.6719	**0.026**	0.223	0.141
N	81.10 ^a^	80.50 ^ab^	78.81 ^b^	80.84 ^a^	0.6764	0.275	0.142	**0.049**
GE	84.24 ^ab^	83.45 ^a^	83.68 ^ab^	84.72 ^b^	0.3920	0.740	0.353	**0.021**

* A total of eight replicates were used per treatment group; SEM, standard error of the mean. ^a b^ mean values within a row with different superscript letters were significantly different.

**Table 7:** The effect of grain preservation method and dietary crude protein levels on small intestinal morphology (least-square means with their standard errors).

	Grain Preservation Method			Crude Protein Level			*p* Values		
	Dried	OA-Preserved	SEM	Standard	Low	SEM	Grain	Protein	Grain × Protein
Duodenum									
VH μm	288.82	307.62	16.567	309.76	286.68	16.297	0.440	0.329	0.810
CD μm	127.41	134.54	6.600	131.90	130.04	6.551	0.466	0.843	0.595
VH:CD	2.38	2.31	0.147	2.41	2.28	0.144	0.755	0.532	0.577
Jejunum									
VH μm	304.50	299.40	18.641	302.81	301.10	18.337	0.852	0.948	0.507
CD μm	124.27	107.77	9.696	111.02	121.03	9.538	0.250	0.468	0.585
VH:CD	2.60	2.85	0.199	2.84	2.61	0.196	0.389	0.430	0.387
Ileum									
VH μm	315.95	295.16	12.932	312.51	298.60	12.722	0.276	0.450	0.157
CD μm	99.16	92.52	4.117	93.42	98.26	4.050	0.275	0.409	0.805
VH:CD	3.24	3.34	0.206	3.44	3.13	0.202	0.746	0.292	0.541

VH, villus height; CD, crypt depth; VH:CD villus height to crypt depth ratio; A total of eight replicates were used per treatment group.

**Table 8:** The effect of grain preservation and dietary crude protein level on the relative expression of genes involved in inflammation that were differentially expressed in the small intestine (least-square means with their standard errors).

	Grain Preservation Method			Crude Protein Level			*p*-Values		
	Dried	OA-Preserved	SEM	Standard	Low	SEM	Grain	Protein	Grain × Protein
Duodenum									
*IL1A*	1.35	0.89	0.152	0.98	1.26	0.152	**0.037**	0.205	0.375
*IL1B*	1.38	1.36	0.287	0.95	1.80	0.287	0.955	**0.046**	0.492
Jejunum									
*IL17*	2.13	0.88	0.405	1.45	1.56	0.405	**0.036**	0.847	0.520
Ileum									
*IL17*	1.45	0.82	0.167	1.10	1.17	0.167	**0.013**	0.773	0.891

*IL1A*, interleukin 1A; *IL1B*, interleukin 1B; *IL17*, interleukin 17; A total of eight replicates were used per treatment group.

**Table 9:** The effect of dietary treatment on the relative abundance of selected bacterial phyla in the ileal and colonic digesta (mean % relative abundance with their standard errors).

Phylum	Treatments *					*p*-Values		
Grain Preservation Method	Dried	OA-Preserved	Dried	OA-Preserved	SEM	Grain	Protein	Grain × Protein
Crude Protein Levels	Standard	Standard	Low	Low				
Ileum								
Firmicutes	92.68	94.57	99.56	91.20	4.989	0.460	0.698	0.244
Colon								
Firmicutes	79.46	77.58	76.98	70.84	3.152	0.197	0.142	0.471
Bacteroidetes	7.46	12.36	8.53	17.63	1.484	**<0.001**	**0.035**	0.325
Actinobacteria	3.59	3.73	4.65	3.65	0.815	0.581	0.516	0.450
Tenericutes	0.50 ^a^	0.17 ^a^	0.48 ^a^	1.71 ^b^	0.462	0.864	0.069	**0.049**
Spirochaetes	0.26	0.11	1.59	0.49	0.446	0.179	**0.031**	0.801

* A total of eight replicates were used per treatment group. ^a b^ Mean values within a row with unlike superscripts letters were significantly different (*p* < 0.05).

**Table 10:** The effect of dietary treatments on the relative abundance of selected bacterial families in the ileal and colonic digesta (mean % relative abundance with their standard errors).

Family	Treatments *					*p* Values		
Grain Preservation Method	Dried	OA-Preserved	Dried	OA-Preserved	SEM	Grain	Protein	Grain × Protein
Crude Protein Content	Standard	Standard	Low	Low				
Ileum								
*Lactobacillaceae*	69.95	87.69	64.15	77.07	3.926	**<0.001**	**0.041**	0.670
*Clostridiaceae*	17.17 ^b^	6.15 ^a^	4.77 ^a^	6.15 ^a^	1.566	0.031	0.001	**0.001**
*Streptocaccaeceae*	5.11 ^a^	0.61 ^b^	0.39 ^b^	4.43 ^a^	1.052	0.743	0.531	**<0.001**
Colon								
*Lactobacillaceae*	19.96	11.19	8.46	6.94	1.689	**0.002**	**<0.001**	0.104
*Lachnospiraceae*	11.92 ^ab^	13.19 ^ab^	16.11 ^a^	10.76 ^b^	1.419	0.355	0.624	**0.017**
*Erysipelotrichaceae*	0.83	0.70	0.35	0.66	0.344	0.621	0.330	0.399
*Eubacteriaceae*	1.22	2.31	3.07	4.29	0.733	**0.049**	**0.003**	0.532
*Ruminococcaceae*	28.73	36.17	37.23	34.12	2.157	0.249	0.111	0.014
*Clostridiaceae*	3.83	2.62	2.59	3.59	0.692	0.894	0.856	0.091
*Propionibacteriaceae*	1.52 ^a^	3.50 ^ab^	4.32 ^b^	3.36 ^ab^	0.786	0.190	0.028	**0.019**
*Streptococcaceae*	0.57	0.37	0.14	0.67	0.288	0.382	0.520	0.137
*Oscillospiraceae*	1.95	1.56	2.06	2.15	0.519	0.719	0.466	0.603
*Spiroplasmataceae*	0.54 ^a^	0.18 ^a^	0.45 ^a^	1.70 ^b^	0.460	0.846	0.090	**0.049**
*Rikenellaceae*	1.57 ^a^	0.68 ^a^	1.22 ^a^	4.51 ^b^	0.751	0.455	0.014	**0.002**
*Hungateiclostridiaceae*	2.75	2.30	1.53	3.22	0.630	0.250	0.608	0.066
*Muribaculaceae*	0.51	0.26	0.32	0.45	0.253	0.775	0.951	0.388
*Acidaminococcaceae*	0.55	0.97	0.59	1.01	0.355	0.198	0.902	0.974
*Veillonellaceae*	0.08	0.62	0.19	0.78	0.313	**0.042**	0.490	0.682
*Prevotellaceae*	5.92	11.63	7.11	13.40	1.294	**<0.001**	0.192	0.863
*Christensenellaceae*	1.69 ^ab^	2.78 ^ab^	3.65 ^a^	1.09 ^b^	0.675	0.184	0.757	**0.003**
*Spirochaetaceae*	0.27	0.12	1.66	0.50	0.456	0.161	**0.029**	0.810

* A total of eight replicates were used per treatment group. ^a b^ Mean values within a row with unlike superscript letters were significantly different (*p* < 0.05).

**Table 11:** The effect of dietary treatment on the relative abundance of selected bacterial genera in the ileal and colonic digesta (mean % relative abundance with their standard errors).

Genus	Treatments *					*p* Values		
Grain Preservation Method	Dried	OA-Preserved	Dried	OA-Preserved	SEM	Grain	Protein	Grain × Protein
Crude Protein Content	Standard	Standard	Low	Low				
Ileum								
*Lactobacillus*	70.56	87.69	66.01	77.75	3.943	**<0.001**	0.071	0.589
*Clostridium*	17.80 ^b^	6.15 ^a^	4.82 ^a^	6.24 ^a^	1.595	0.025	<0.001	**<0.001**
*Streptococcus*	2.09 ^a^	0.61 ^a^	0.39 ^a^	8.51 ^b^	1.305	0.064	0.326	**<0.001**
Colon								
*Lactobacillus*	20.20	11.22	8.75	7.01	1.699	**<0.001**	**<0.001**	0.114
*Collinsella*	1.96	0.20	0.46	0.16	0.529	**0.018**	0.216	0.359
*Anaerobutyricum*	0.08	0.53	0.20	0.06	0.256	0.741	0.584	0.152
*Catenibacterium*	0.26	0.04	0.15	0.13	0.182	0.401	0.813	0.469
*Gemmiger*	8.87	5.80	7.09	5.27	1.053	**0.015**	0.261	0.649
*Ruminococcus*	2.41	0.76	2.14	1.21	0.517	**0.009**	0.574	0.347
*Faecalibacterium*	15.26 ^a^	24.88 ^b^	24.44 ^b^	24.63 ^b^	1.755	0.003	0.006	**0.004**
*Butyricicoccus*	1.89	1.33	1.03	0.81	0.486	0.375	0.103	0.868
*Holdemanella*	1.16	0.19	0.20	0.25	0.381	0.254	0.289	0.151
*Clostridium*	1.97 ^ab^	1.28 ^ab^	0.85 ^a^	2.69 ^b^	0.580	0.248	0.879	**0.016**
*Streptococcus*	0.57	0.37	0.14	0.66	0.288	0.390	0.519	0.136
*Oscillibacter*	1.95	1.55	2.13	2.16	0.519	0.678	0.417	0.647
*Spiroplasma*	0.54 ^a^	0.18 ^a^	0.46 ^a^	1.71 ^b^	0.462	0.853	0.087	**0.049**
*Anaerocella*	1.56 ^ab^	0.69 ^a^	1.22 ^ab^	3.19 ^b^	0.631	0.827	0.052	**0.009**
*Pseudobutyrivibrio*	0.14	0.48	0.46	0.71	0.297	0.207	0.240	0.550
*Eubacterium*	1.22	2.31	3.16	3.45	0.702	0.149	**0.001**	0.270
*Dorea*	1.26 ^a^	2.71 ^ab^	4.14 ^b^	1.10 ^a^	0.720	0.306	0.598	**<0.001**
*Prevotella*	4.63 ^a^	10.71 ^b^	6.30 ^a^	7.70 ^ab^	1.157	<0.001	0.933	**0.027**
*Phascolarctobacterium*	0.55	0.91	0.58	0.65	0.338	0.497	0.753	0.662
*Roseburia*	1.64	4.08	1.72	2.68	0.715	**0.009**	0.448	0.335
*Fournierella*	0.48	0.71	0.58	0.96	0.369	0.327	0.591	0.910
*Megasphaera*	0.02	0.07	0.13	0.49	0.247	0.422	0.230	0.951
*Agathobacter*	0.81	1.61	1.11	1.34	0.448	0.198	0.832	0.459
*Blautia*	2.31	1.04	3.13	1.09	0.626	**0.003**	0.541	0.659
*Christensenella*	1.48 ^ab^	2.78 ^a^	2.14 ^ab^	1.09 ^b^	0.589	0.932	0.319	**0.027**
*Pseudoflavonifractor*	1.25	0.54	1.77	0.68	0.503	**0.028**	0.465	0.880
*Hungateiclostridium*	0.31	0.18	0.14	0.07	0.197	0.563	0.385	0.938
*Treponema*	0.17	0.11	1.48	0.46	0.430	0.306	**0.029**	0.622
*Dialister*	0.05	0.02	0.06	0.11	0.127	0.949	0.616	0.664

* A total of eight replicates were used per treatment group. ^a b^ Mean values within a row with unlike superscript letters were significantly different (*p* < 0.05).

**Table 12:** The effect of dietary treatment on the molar and total concentrations of VFA in mmol/g of digesta in the colon (least-square means with their standard errors).

	Treatment *					*p* Values		
Grain Preservation Method	Dried	OA-Preserved	Dried	OA-Preserved	SEM	Grain	Protein	Grain × Protein
Crude Protein Level	Standard	Standard	Low	Low				
Colon								
Acetate	0.499	0.512	0.422	0.423	0.0161	0.667	**<0.001**	0.735
Propionate	0.281	0.281	0.332	0.339	0.0079	0.643	**<0.001**	0.666
Butyrate	0.161	0.145	0.190	0.184	0.0120	0.358	**0.008**	0.689
Valerate	0.038	0.032	0.033	0.026	0.0044	0.145	0.235	0.944
Isobutyrate	0.011	0.015	0.012	0.015	0.0021	0.103	0.703	0.977
Isovalerate	0.011	0.017	0.012	0.013	0.0024	0.126	0.568	0.352
BCFA	0.059	0.054	0.063	0.052	0.0057	0.168	0.846	0.666
Total	220.05 ^a^	207.18 ^a^	162.47 ^b^	196.79 ^a^	10.5124	0.316	0.003	**0.033**

BCFA, branched-chain fatty acids. * A total of eight replicates were used per treatment group. ^a b^ Mean values within a row with unlike superscript letters were significantly different (*p* < 0.05).

## Data Availability

The data presented in this study are available on request from the corresponding author.
